# PKCa Agonists Enhance the Protective Effect of Hyaluronic Acid on Nitric Oxide-Induced Apoptosis of Articular Chondrocytes *in Vitro*

**Published:** 2013-12

**Authors:** Jian-lin Zhou, Hong-song Fang, Hao Peng, Qiong-jie Hu, Shi-qing Liu, Jiang-hua Ming, Bo Qiu

**Affiliations:** 1 Department of Orthopedics, Renmin Hospital of Wuhan University, Wuhan, 430060, Hubei Province, People’s Republic of China; 2 Department of Radiology, Tongji Hospital, Tongji Medical College, Huazhong University of Science and Technology, Wuhan 430030, Hubei Province, People’s Republic of China

**Keywords:** Apoptosis, Nitric Oxide, PKC-α, PMA

## Abstract

***Objective(s):*** Protein kinase C (PKCα) is involved in modulating articular chondrocytes apoptosis induced by nitric oxide (NO). Hyaluronic acid (HA) inhibits nitric oxide-induced apoptosis of articular chondrocytes by protecting PKCα, but the mechanism remains unclear. The present study was performed to investigate the effects and mechanisms of PKCα regulate protective effect of hyaluronic acid.

***Materials and Methods*** The ratio of apoptotic cell and cell viability was surveyed by PCNA and MTT assay. The expression of caspase-3 was determined by real-time PCR and western blot.

***Results:*** It was showed that HA was able to reduce the nuclei fragment and PCNA expression, and NO-induced articular apoptosis blocked by HA, pretreated chondrocytes with PMA, HA significantly inhibits the activation of caspase-3 induced by NO, but pretrement with CHR, HA significantly incresed the expression of caspase-3.

***Conclusion:*** The results may be showed that PKCa regulate the expresion of caspase-3, which contribute to the apoptosis of chondrocytes induced by NO. PKC α agonists enhance the protective effect of hyaluronic acid on nitric oxide-induced articular chondrocytes apoptosis.

## Introduction

Apoptotic death of articular chondrocytes is a central feature in Osteoarthritis (OA) cartilage degeneration. Chondr-ocytes are essential for maintaining the normal matrix of cartilage, and chondrocyte apoptosis plays an important role in the pathogenetic pathway of osteoarthritis, since apoptotic bodies may remain in the extracellular matrix of cartilage due to the absence of phagocytes and may accelerate matrix degradation by releasing degradative enzymes (1). Therefore, the apoptosis of chondrocytes seems to be a potential target for therapeutic interventions in OA. 

Nitric oxide (NO) is a highly reactive free radical synthesized from the L-arginine by the members of the nitric oxide synthase family including iNOS. It has also long been considered to be a catabolic factor that contributes to the OA disease pathology by mediating a number of processes, including apoptos-is, suppressing the synthesis of the cartilage matrix and increasing the expression of proinflammatory cytokines (2-4). High concentrations of nitrites and nitrates have been found in the synovial fluid and plasma of patients with arthritis. NO cause articular chondrocytes apoptosis by the inhibition of PKCα (5-7). Our previous studies also indicate that SNP-treated chondrocytes cause inhitition of PKCα, which may cause chondrocytes apoptosis in our culture system. 

Hyaluronic acid, which is a major natural compo-nent in the cartilage extracellular matrix, has a protective effect on the progression of OA (8-13). We have previously shown that HA inhibit the chondrocytes apoptosis and dedifferentiation in a dose-dependent manner (14). And the inhibitory effects of HA on apoptosis are derived from their ability to block NO-induced inhibition of PKCα. Nevertheless, the regulation mechanism of PKCα remains unclear and, at present, few data is available on its effect on chondrocyte apoptosis induced by NO. The aim of this study was to investigate the effects and mechanisms of PKCα agonists or antagonist regulate the profective of HA on nitric oxide-induced articular chondrocytes apoptosis *in vitro*.

## Materials and Methods

***Materials***

Hyaluronic acid (molecular weight: 500–730 kDa) was provided by Worthington (Lakewood, NJ). Trypsin, Dulbecco's Modified Eagle's Medium (DMEM)/F12, and fetal bovine serum were from Gibco BRL. Hoechst 33258 was obtained from Beyotime Institute of Biotechnology (China). Phorbol -12-myristate-13-acetate (PMA), Chelerythrine (CHR) and all another chemicals used were of the highest grade available commercially. 

***Articular chondrocytes*** ***preparation***

Primary cultures were prepared as described previously. After removal of non-chondrocytes in a preplating step, and Articular chondrocytes were plated on culture dishes at a density of 5×10^4^ cells/cm^2^. The medium was replaced every 2 days after plating, and cells reached confluence at 4 days of culture. the cells were exposed to 5 different control or treatment protocols groups: 1) control; 2) SNP(1.0 mM); 3）SNP plus HA; 4)PMA (100 nM) plus HA (100 μg/ml) plus SNP; 5) the PKC antagonist chelerythrine (CHR; 10μg/ml) plus HA plus SNP; After 1 hr pretreatment with agonists in the presence or absence of antagonists, the cells were incubated with SNP, HA or medium alone (control group) for 23 hr (37°C; 95% oxygen/5% CO_2_) and were then washed with phosphate-buffered normal saline and prepared for evaluation of cell survival.

***Determination of cell viability***

Cell viability was assessed by using the 3-(4,5-dimethylthiazol-2-yl)-2,5 diphenyltetrazdium bromi-de (MTT) assay. Briefly, chondrocytes were seeded at 5×10^4^/100 μl/well in 96-well microtiter plates. Cell apoptosis was induced by treating with 1 mM SNP. To protect cells, different doses of HA (0, 50, and 100 μg/ml) were added to chondrocytes 1 hr before being treated with 1 mM SNP. MTT was then added to each well to a final concentration of 0.125 mg/ml after treatment for 24 hr, and the plate incubated at 37°C for 4 hr, after which the formazan product was solubilized with 100 μl dimethylsulfoxide (DMSO) and the optical density was read at a wavelength of 595 nm. 

***Hoechst 33258 staining***

Changes in nucleic morphology of apoptotic cells were detected by staining with the DNA-binding fluorochrome Hoechst 33258. Cells were grown on glass coverslips to subconfluence and treated with test components as described above in DMEM medium and then fixed with 4% paraformaldehyde, and then were loaded with 2% Hoechst 33258 (final concentration) in DMEM/F12 for 30 min at room temperature. After the cells were washed twice with phosphate-buffered saline, the nuclear condensation and fragmentation were observed under a fluore-scence microscope (Olympus) with excitation at 355 nm and emission at 465 nm. And were mounted onto slides using fluorescent mounting medium (Dako, Carpinteria, CA, USA). The proportion of cells undergoing apoptosis 24 hr after initiation of treatment was determined by counting the total number of cells and the cells exhibiting two or more membrane blebs and brightly stained condensed and fragmented chromatin per high power field. Apoptotic index was calculated as the percentage of cells displaying the characteristics described above. A minimum of five different fields and at least 300 cells for each treatment were counted for each sample. All experiments were repeated three times to ensure reproducibility.

***Imunocytochemical study for proliferating cell nuclear antigen (PCNA)***

Briefly, chondrocytes endogenous peroxidase was blocked by incubation in 3% H_2_O_2_-methanol for 3 min. After washing three times in phosphate-buffered saline (PBS), the sections were incubated with monoclonal antirat PCNA for 30 min at room temperature at a dilution of 1:100 in 0.05 *M *Tris buffer, pH 7.6, with 0.1% NaN3, and then washed three times in PBS. The second antibody was applied at a dilution of 1:500, and incubated for 10 min at room temperature. Streptavidin-peroxidase was then applied to the sections in 0.05 M Tris buffer for 10 min at room temperature. Slides were developed, following washing in PBS three times, using 3.3% diaminobenzidine in 50 mM Tris buffer, pH 7.6. The sections were counterstained with Mayer’s hematoxylin for 30 sec at room temperature, and mounted in glycerin gel. Omission of the primary antibody (anti-PCNA antibody) served as a negative control.

***Real-time PCR analysis***

The total RNA was extracted using the Trizol Reagent (Invitrogen) according to the manufacturer’s protocol. The RNA samples were quantified by A260. Then RNA was reverse-transcribed to cDNA using an RT-PCR kit (Takara, Dalian, China) according to the manufacturer’s protocol. The cDNA was analyzed immediately or stored at −20°C. Quantitative PCR amplifi cation was performed by an ABI 7900HT fast real-time PCR system (Applied Biosystems, Foster City, CA, USA), and the SYBR Green I fluorescent dye method was used to quantify cDNA. PCR cycling conditions consisted of an initial denaturing step for 10 sec at 95°C; then 40 cycles of 5 sec each at 95°C; followed by 30 sec at 60°C. A stable, reliable standard curve was established using synthesized oligonuc-leotides resembling cDNA fragments in five fold decrements as template. The β-actin from the same sample was used as the internal control. The relative contents of the copy numbers of the target gene’s mRNA were then calculated, through which we could determine the gene expression level and its trend of change. The specifi city of each reaction was controlled by melting curve analysis. A negative PCR control containing water instead of cDNA was prepared. Real-time PCR was conducted in triplicate in three independent experiments. The sequences were as Table 1. 

**Table 1:** Oligonucleotide primers for real-time polymerase chain reaction analysis of cartilage matrix gene expression

Gene description	Forward primer sequence	Reverse primer sequence
β-actin	GGAGATTACTGCCCTGGCTCCTA	GACTCATCGTACTCCTGCTTGCTG
Caspase-3	CCTTGCCTCAAACAAAGGTT	CCTGAGTGGGTTCACAATGTT

***Western blot***

Western blotting assay cell lysis was carried out using M-PER mammalian protein extraction reagent (Pierce) and protease inhibitor cocktail set III (Calbiochem) plus 5 mmol/l EDTA. A total volume of 20 µg of protein was separated by 10% SDS-PAGE. The separated proteins were transferred to a polyvinylidene difluoride membrane. After being blocked with 5% non-fat milk in Tris-buffer saline containing 0.05% Tween 20 (TBST), the membrane was incubated with the corresponding antibodies (Santa Cruz) in TBST overnight at 4°C. The membrane was then washed 3 times with TBST and incubated with the horseradish peroxidase-conjugated secondary antibody (1:5000) in TBST for 2 hr at room temperature and visualized using ECL kit. The relative amount of immunoreactive protein in each sample was quantitated by densitometry (AMBIS Radioanalytic and visual imaging system, Ambis Inc.). The densitometric data from each blot normalized to the control condition with the value set equal to one for each experiment. All tests were repeated two times.

***Statistical analysis***

All data were expressed as mean ± standard deviation (SD). Statistical analysis was performed using a one-way ANOVA and a *P-* value of less than 0.05 was considered statistically significant.

## Results

***PMA enhances HA protective effect of chondrocytes from NO-induced cell death***

**Figure 1:**
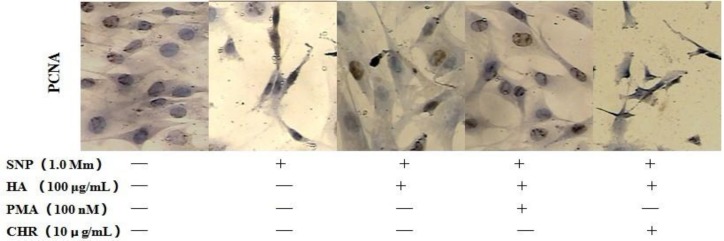
Nitric oxide (NO) induces rat chondrocytes apoptosis by inhibiting the PCNA expression *in vitro*. Fluorescence micrographs of rat chondrocytes following treatment with the NO donor SNP (sodium nitroprusside, 1 mM) for 24 hr, PCNA expression was detected by imunocytochemical study. Pretreated chondrocytes with PMA, HA significantly increase the activation of PCNA induced by NO, but CHR and HA significantly inhibit the expression of PCNA

Our previous studies have showed HA blocks NO-induced apoptosis of primary culture. In this study, we pretreated cell with agonists in the presence or absence of antagonists, and examined apoptotic cell by imunocytochemical staining for Proliferating Cell Nuclear Antigen (PCNA) (Figure 1). As shown in Figure 1, HA blocked chondrocyte apoptosis and PMA can augment PCNA expression, and CHR significantly inhibited the chondrocytes survival. 

***Effects of PMA on nucleic morphology in NO-induced chondrocytes***

**Figure 2:**
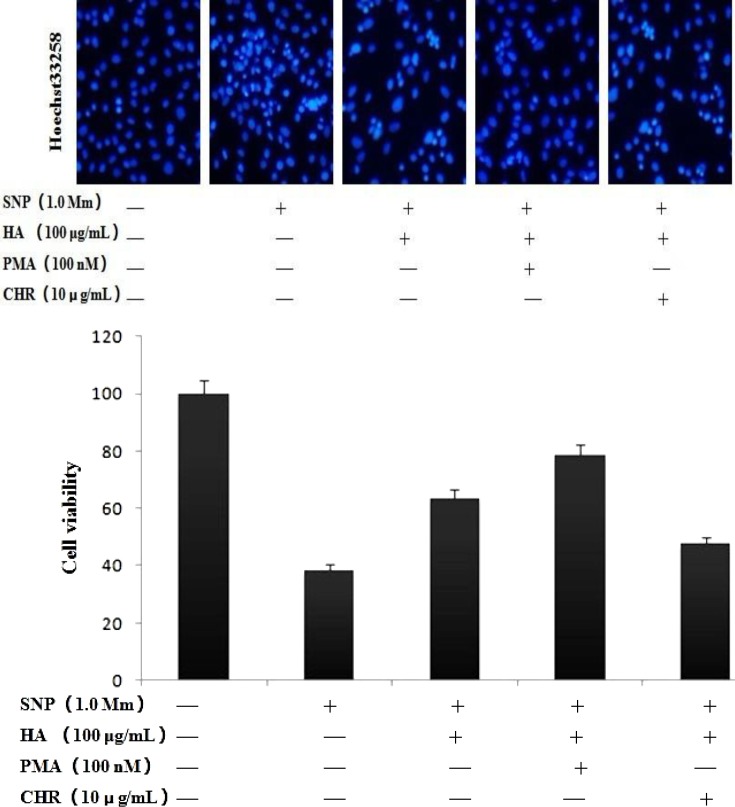
Hoechst 33258 analysis the nucleic morphologic changes of apoptotic chondrocytes, NO cause nuclei condensed, and fragmented and cell apoptosis. Pretreated chondrocytes with PMA, HA significantly increase the cell viability, but pretement with CHR, HA significantly inhibit the chonrocytes survival. *P<0.05 vs. sham group, ** P<0.05 vs. Control group

To analyze the nucleic morphologic changes of apoptotic chondrocytes, cells were stained with Hoechst 33258 (Figure 2 upper) and the reduction of cell viability determined by MTT assay (Figure 2 bottom). As shown in (Figure 2 upper), the nuclei of control chondrocytes had a homogeneous pattern of staining. In NO-induced chondrocytes, a considerable proportion of cells showed typical characteristics of apoptosis with condensed, fragmented nuclei. Pretreatment with PMA, HA caused a significant decrease in NO-induced apoptotic cells. HA blocked chondrocyte apoptosis and PMA can augment protective effect, and CHR significantly inhibited the chondrocytes survival.

***Effect of PMA on NO-induced apoptosis of chondrocytes***

**Figure 3:**
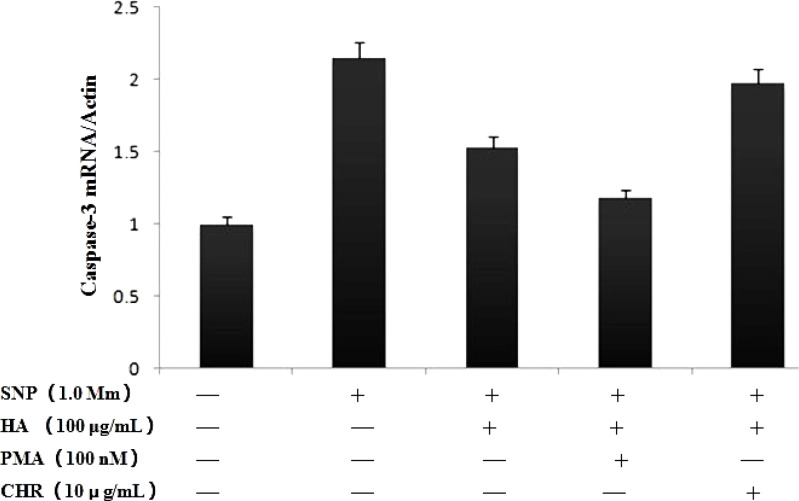
Changes of caspase-3 mRNA expression was deteacted by real time PCR. NO increased the mRNA expression of caspase-3, Pretreated chondrocytes with PMA, HA significantly inhibit the caspase-3 mRNA expression, but pretrement with CHR, HA could not decrease the caspase-3 mRNA expression. *P<0.05 vs. Sham group. ** P<0.05 vs. control group

**Figure 4:**
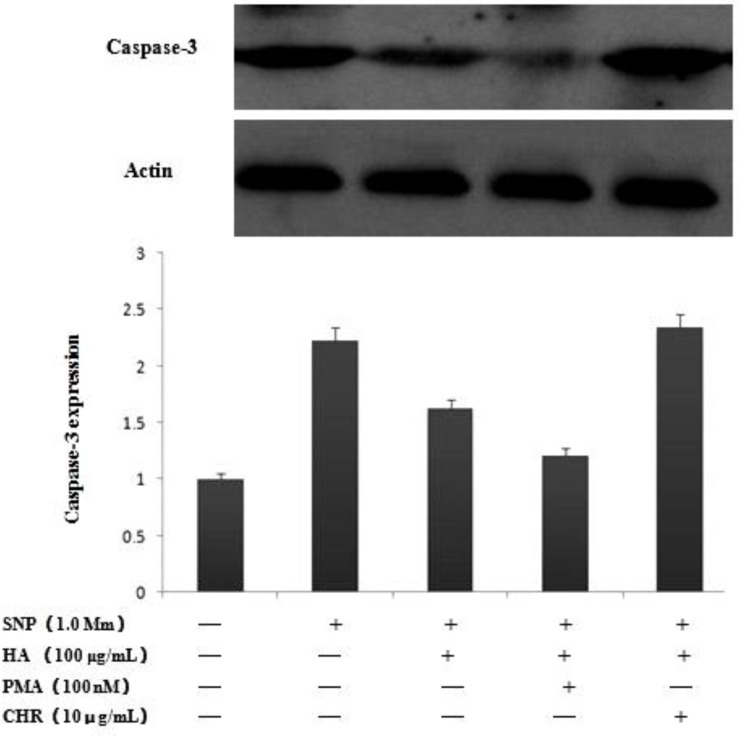
Expression of casapase-3 by Western blot. Articular chondrocytes were pretreated with PMA, CHR and then treated by HA for 1 hr and then exposed to 1 mM SNP for 24 hr. HA inhibits the casapase-3 induced by SNP, PMA can enhanced this trend, but pretement with CHR, HA play no protective effect. The data represent mean values with S.D. and results of a typical experiment selected from at least five independent experiments. *P<0.05 vs. sham group, ** P<0.05 vs. control group

Real-time PCR and western blot revealed significant differences in the expression of caspase-3. NO production caused activation of caspase-3. As shown in Figure 3 and 4, NO causes aggrecan and type caspase-3 mRNA and protein expression increased, pretreatment of chondrocytes with HA blocked the NO-induced increase in caspase-3 expression. Pretreatment with PMA, HA significantly inhibits the activation of caspase-3 induced by SNP, but CHR significantly increased the expression of caspase-3. 

## Discussion

Chondrocytes are essential for maintaining the normal matrix of cartilage, and the major feature of OA is a progressive loss of articular cartilage. Apoptotic death of articular chondrocytes is a central feature in OA cartilage degeneration. Apoptosis is important for the clearance of damaged or variant cells, and alterations in apoptosis contribute to the pathway of osteoarthritis. Since apoptotic bodies may remain in the extracellular matrix of cartilage due to the absence of phagocytes and may accelerate matrix degradation by releasing degradative enzymes (3). Therefore, the apoptosis of chondrocytes seems to be a potential target for therapeutic interventions in OA. 

Key regulators of the apoptotic pathway include pro- and antiapoptotic members of the Bcl-2 family, caspase proteases, which cleave cellular proteins, and the family of IAP proteins, which regulate the activity of activated caspases. Serine/threonine protein kinases are also known to regulate apoptosis, including the phosphoinositide 3-kinase/AKT pathway, members of the mitogen-activated protein kinase family (MAPKs), and the protein kinase C (PKC) pathway (15).

Nitric oxide (NO) is synthesized by conversion of L-arginine to NOH-arginine and ultimately to L-citrulline plus NO Osteoarthritic chondrocytes produce a number of inflammatory mediators including IL-1β, TNF-á, prostaglandins and NO (16).

NO mediates the effects of a number of proinflammatory cytokines, including IL-1β and TNF-á. *In vivo* experiments using a canine osteoarthritis model demonstrated that stimulation of chondrocytes by NO is responsible in part for the subsequent upregulation of IL-18 synthesis as well as the synthesis of interleukin-1-converting enzyme (ICE), a caspase required for maturation of both IL-1β and IL-18 (16, 17).

NO has been reported to be a key inducer of chondrocyte apoptosis, a central pathogenic feature of OA. It has been suggested that either endogenous or exogenous NO can induce apoptosis in chondrocytes via a mitochondriadependent mechanism (18). Human chondrocytes are exposed to either exogenous NO via incubation with sodium nitroprusside (SNP) or endogenous NO via incubation with lipopolysccharide (LPS) and interferon (IFN)-a, NO, ROS and cytochrome C levels all increase, as does caspace-3 activation and DNA fragmentation, all hallmarks of apoptosis (19, 20).

High concentrations of nitrites and nitrates have been found in the synovial fluid and plasma of patients with arthritis. In our results show in Figure 1 (PCNA, Hochest 33258 and MTT analysis), we could find that NO induce chondrocyte apoptosis by inhibiting PCNA expression. NO cause apoptosis of articular chondrocytes by the activation of ERK-1/2 and p38 kinase and inhibition of PKCα and-ζ (5-7). Our current results indicate that SNP-treated chondrocytes cause inhitition of PKCα, which may cause chondrocytes apoptosis in our culture system.

Hyaluronic acid, which is a major natural component in the cartilage extracellular matrix, has a protective effect on the progression of OA. We have previously confirmed that HA decrease iNOS expression in synovium and NO content in synovial fluid of rabbits with traumatic osteoarthritis (21), it also inhitited apoptosis induced by NO by blocking the NO-induced inhibition of PKCα in dose-dependent manner (22). In the present studies, we have used an activation and inhibitory form of PKCa to explore the function of PKCa in the apoptotic pathway.

The PKC family consists of 11 isoforms, with individual isoforms exhibiting varying substrate specificity, as well as differences in their subcellular localization and response to specific stimuli. The role of PKC in apoptosis is controversial, with data supporting both pro- and antiapoptotic functions. In the previous and current studies, we have examined the role of PKCa, a PKC isoform associated with proliferation in chondrocytes, as show in Figure 1 and 2, NO inhibited chondrocytes PCNA expression, induced nuclei fragment and apoptosis. HA can restore PCNA expression and blocked chondrocyte apoptosis. Activation of PKCa by PMA can augment protective effect, but inhibition by CHR significantly increased the chondrocytes apoptosis. 

We have previously reported that NO increased caspase-3 expression and induced chondrocytes apoptosis by inhibited PKCa, and HA could block this tendency (14). In current studies, we confirm that activation and inhibitory form of PKCa to explore the function of PKCa in the apoptotic pathway, we pretreated chondrocytes with PMA, HA significantly inhibits the activation of caspase-3 induced by SNP, but pretreat with CHR, HA significantly incresed the expression of caspase-3. The results may be showed that PKCa agonists inhibited the expresion of caspase-3, which contribute to the apoptosis of chondrocytes.

## Conclusion

 PKC α agonists enhance the protective effect of hyaluronic acid on nitric oxide-induced articular chondrocytes apoptosis.
